# Middle Ear Surgeries for Chronic Otitis Media Improve Cognitive Functions and Quality of Life of Age-Related Hearing Loss Patients

**DOI:** 10.3389/fnins.2022.786383

**Published:** 2022-02-17

**Authors:** Juanjuan Gao, Junyan Chen, Jia Xu, Sichao Liang, Haijin Yi

**Affiliations:** Department of Otolaryngology, Head and Neck Surgery, Beijing Tsinghua Changgung Hospital, School of Clinical Medicine, Tsinghua University, Beijing, China

**Keywords:** middle ear surgeries, chronic otitis media, age-related hearing loss, cognition functions, quality of life

## Abstract

Age-related hearing loss (ARHL) may limit communication, which is closely associated with cognitive decline of the elderly and negatively affects their quality of life. In ARHL patients who suffer chronic otitis media (COM), hearing impairment may worsen and negatively affect the cognition and quality of life. It is currently unknown whether restoration of the conductive hearing in the mixed hearing loss through middle ear surgeries can improve both the cognitive function and quality of life of the ARHL patients. Therefore, in the present study, the ARHL patients were followed up for 6 months after middle ear surgeries for COM, and both the cognitive functions and quality of life of the patients were assessed using Montreal Cognitive Assessment and Glasgow Benefit Inventory. It was found that both the cognitive functions and quality of life were improved 6 months after middle ear surgeries. In conclusion, hearing recovery after middle ear surgeries could improve cognitive functions and quality of life of ARHL patients with COM, and surgical intervention is, hence, recommended for COM.

## Introduction

With global aging, the number of age-related diseases is increasing. According to the WHO, hearing loss is the second cause of disability throughout the world, over 42% of which is age-related hearing loss (ARHL) (WHO, Deafness and hearing loss, 2021). ARHL, which is also called presbycusis and characterized by progressive bilateral, symmetrical, sensorineural hearing loss, may limit communication and has a close relationship with cognitive decline and even dementia (Fortunato et al., 2016; Griffiths et al., 2020), negatively affecting the quality of life. However, the exact mechanisms related to the relationship between hearing loss and cognitive impairment are still unclear. One of the mechanisms is that the impoverished environment caused by auditory deprivation can negatively change brain structure and function, leading to cognitive impairment (Peelle et al., 2011; Mamo et al., 2019; Griffiths et al., 2020; Häggström et al., 2020).

To date, the treatment of hearing impairment in senile population is hearing aids and cochlear implantation. Cochlear implantation is indicated for patients with severe to profound sensorineural hearing loss who cannot benefit from hearing aids (Clark et al., 2012). Moreover, among the elderly with hearing impairment who are indicated for hearing aid intervention, two thirds did not wear hearing aids (Fischer et al., 2011). According to the Global Burden of Disease in 2019, over 65% of the elderly aged above 60 years experienced hearing impairment. Furthermore, it has been shown that the prevalence of chronic otitis media (COM) is 10.5% in China in those aged more than 60 years (Ren and Chen, 2016). In the meantime, many old people with presbycusis may also suffer from COM that can ultimately result in mixed hearing loss (i.e., combination of conductive hearing loss and presbycusis), which makes hearing of the elderly much worse. In ARHL patients who also suffer COM, they mainly present with symptoms of hearing loss, recurrent otorrhea, and tinnitus, and it may be more inconvenient to wear hearing aids, especially when otorrhea occurs. Under both ARHL and COM, the hearing impairment may worsen, and thus, negatively affect the cognition and quality of life.

However, it is currently unknown whether correction of the conductive hearing in the mixed hearing loss through middle ear surgeries can improve both the cognitive function and quality of life of the ARHL patients.

Therefore, the aim of the present study was to investigate whether middle ear surgeries could improve the cognitive functions and quality of life in elderly patients who suffered both ARHL and COM, and to further investigate the mechanisms related to the relationship between hearing loss and cognitive impairment.

## Subjects and Methods

### Subjects

Patients aged over 60 years who were diagnosed with both ARHL and COM and underwent tympanoplasty under general anesthesia by experienced otologists in our department from January 2015 to December 2020 were included in this study. The purpose of the study was explained to the patients, and informed consents were obtained from all the participants. The protocol was approved by the Ethics Committee of Beijing Tsinghua Changgung Hospital.

Exclusion criteria: (1) patients who were not benefited from middle ear surgeries [i.e., improvement of the hearing thresholds less than 15 dB or improvement of the maximum speech discrimination score (SDSmax) less than 10%]; (2) patients who had moderate, severe, or profound ARHL and are not benefited from middle ear surgeries; (3) patients who suffered active COM or were complicated by cholesteatoma; (4) patients who had history of middle ear surgeries or were combined with middle ear tumors; (5) patients who had received hearing aids before; (6) patients who had visual impairment that limited the cognitive test; and (7) patients who suffered from diseases that may interfere the cognition or quality of life during the 6-month follow-up after operation, such as cerebrovascular disease, craniocerebral trauma, schizophrenia, sudden hearing loss, severe tinnitus, etc.

Patients included in our study were examined by the same audiologist. Pure tone audiometry (PTA), SDSmax tests, and temporal bone high-resolution CT (HRCT) were carried out before operation and 6 months after operation, respectively. The average hearing threshold was calculated based on all PTA frequencies at the surgical side. Hearing loss was categorized based on the classification proposed by the WHO (World report on hearing, 2021): normal hearing, less than 20 dB HL; mild hearing loss, 20–35 dB HL; moderate hearing loss, 35–50 dB HL; moderately severe hearing loss, 50–65 dB; severe hearing loss, 65–80 dB HL; profound hearing loss, 80–95 dB HL; and complete or total hearing loss, 95 dB or greater.

### Intervention

The continuity of the ossicular chain was checked by temporal bone HRCT before operation. During operation, we attempted to preserve the ossicular chain if the mobility was good (type I tympanoplasty). Ossicular chain reconstruction during tympanoplasty was performed when the incus was eroded (partial ossicular replacement prosthesis, PORP) or the stapes was eroded (total ossicular replacement prosthesis, TORP). Perforation of the tympanic membrane was repaired using a fascia graft and/or tragus cartilage.

### Outcomes

Cognitive performance was assessed using Montreal Cognitive Assessment (MoCA), by a separate observer. The MoCA consists of seven subsections, including Visuospatial/Executive, Naming, Memory, and Delayed Recall, Attention, Language, Abstraction, and Orientation. According to the answers, the total score ranges from 0 to 30.

Quality of life was assessed using Glasgow Benefit Inventory 9. Glasgow Benefit Inventory is a kind of questionnaire covering 18 questions, which addresses changes in quality of life after operation. The scores for each question range from 1 to 5: 1, the worst change; 5, the best change; and 3, no change. The numerical data from the questionnaire were then converted to a GBI index score ranging from −100 (the worst outcome) to +100 (the best outcome). GBI consists of three subscales: general subscale scores, social support scores, and physical health scores. The Glasgow Benefit Inventory is a systematic review of the use and value of an otorhinolaryngological generic patient-recorded outcome measure.

The MoCA assessment was performed before middle ear surgeries and 6 months after operation, respectively, and the scores were compared. The GBI assessment was performed 6 months after operation.

### Statistics

Quantitative data were expressed as mean ± standard deviation (SD). The parametric data were compared using the paired *t*-test, and *p* < 0.05 was considered statistically significant. Concerning multivariate analysis for improvement of the total MoCA scores and the seven subsection scores, *p* < 0.025 was considered statistically significant (one-tailed test). Pearson’s correlation analysis was performed to determine the correlation between hearing improvement and MoCA/GBI. SPSS 22.0 program (SPSS Inc., Chicago, IL, United States) and GraphPad Prism 6.05 software (GraphPad Software, Inc., La Jolla, United States) were used for statistical analysis.

## Results

### Demographic and Treatment of the Patients

A total number of 29 patients, including 23 females and six males, were enrolled in the study. The age of the subjects ranged from 60 to 74 years, with a mean age of 65.6 ± 4.7 years. All patients were diagnosed with ARHL and COM, and underwent tympanoplasty. The demographic features and treatments of the subjects are shown in Table 1.

**TABLE 1:** Demographics properties and treatment for all patients included.

Demographics properties and treatment for patients	

Parameters	*n*
Age in years (mean ± SD)	65.6 ± 4.7
Sex	
Female	23
Male	6
ARHL with COM	
Right ear	18
Left ear	11
Treatment	
Type I tympanoplasty	20
Tympanoplasty with PORP	8
Tympanoplasty with TORP	1

### Surgery Outcomes of the Patients

**FIGURE 1:**
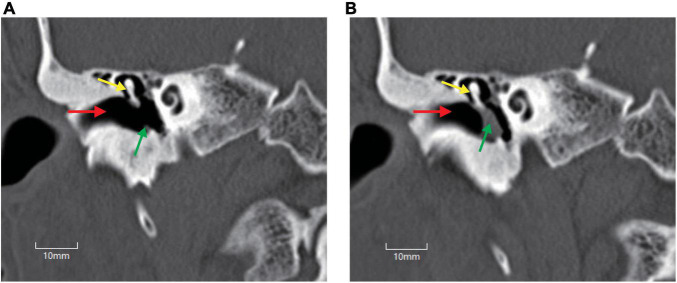
Temporal bone high-resolution CT (HRCT) before and after operation. **(A)** HRCT before operation. **(B)** HRCT after operation. Perforation of the tympani membrane (green arrow in **A**) was repaired using a tragus cartilage (green arrow in **B**), and the middle ear was reconstructed to a normal anatomical structure to the maximum extent. Red arrow, external auditory canal; yellow arrow, malleus.

**FIGURE 2:**
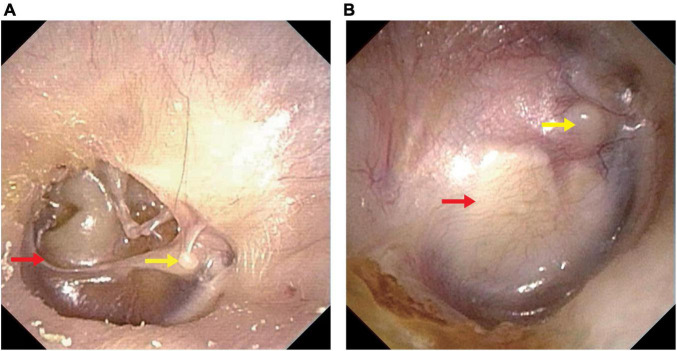
Endoscopic pictures before and after operation. **(A)** Endoscopic picture before operation. **(B)** Endoscopic picture after operation. Perforation of the tympani membrane (red arrow in **A**) was repaired using a tragus cartilage (red arrow in **B**) and healed well. Yellow arrow, lateral process of the malleus.

To intuitively evaluate the surgery outcomes, all patients underwent temporal bone HRCT before and after operation. Tympanoplasty in the present study could reconstruct the middle ear into a normal anatomical structure to the maximum extent (Figures 1, 2).

The preoperative and postoperative hearing function is shown in Table 2. The decrease in air–bone–gap (ABG) (dB) at 6 months after operation was found to be statistically significant (Table 2).

**TABLE 2:** Comparison of PTA preoperation and 6 months postoperation.

	Preop (dB)	Postop (dB)	*P*
BC threshold	35.1 ± 6.7	34.9 ± 6.3	0.2144
AC threshold	71.3 ± 13.6	41.5 ± 8.3	<0.0001
ABG	36.2 ± 10.9	6.6 ± 4.4	<0.0001
Hearing gain		29.6 ± 8.1	

*Paired t test. Means ± SD. SD, Standard deviation; BC, bone conduction; AC, air conduction; ABG, air–bone–gap; Hearing gain, postoperative ABG – preoperative ABG.*

The preoperative and postoperative SDSmax is shown in Table 3. The improvement in SDSmax at 6 months after operation was found to be statistically significant (Table 3).

**TABLE 3:** Comparison of SDSmax preoperation and 6 months postoperation.

	Preop (%)	Postop (%)	*P* value
SDSmax	73.9 ± 21.0	94.0 ± 4.8	<0.0001
SDSmax gain		20.1 ± 17.1	

*Paired t test. Means ± SD. SDSmax gain, postoperative SDSmax – preoperative SDSmax; Preop, preoperation; Postop, postoperation.*

### The Preoperative and Postoperative MoCA Total Scores Were Significantly Different

The MoCA assessment was performed before operation and 6 months after operation, respectively. The preoperative and postoperative MoCA total scores and subsection scores were compared (Table 4). Concerning the total MoCA scores, before operation, the mean score was 21.9 ± 4.7, and it increased to 23.9 ± 4.1 at 6 months after operation, with significant difference (*p* < 0.0001). As shown in Table 3, of the subsections, the language capacity was significantly improved (*p* < 0.0001). Furthermore, the Visuospatial/Executive, Memory and Delayed Recall, and Attention were also improved, with *p* < 0.05.

**TABLE 4:** Comparison between preop and postop MoCA scores.

	Preop	Postop	*P* value	*P* value summary
Total	21.9 ± 4.7	23.9 ± 4.1	<0.0001	****
Visuospatial/Executive	2.9 ± 1.3	3.1 ± 1.2	0.0116	*
Naming	2.4 ± 0.6	2.5 ± 0.6	0.0831	ns
Memory and delayed recall	2.8 ± 1.3	3.1 ± 1.4	0.0173	*
Attention	5.2 ± 1.2	5.3 ± 1.0	0.0433	*
Language	1.9 ± 0.6	2.7 ± 0.5	<0.0001	****
Abstraction	1.1 ± 0.6	1.2 ± 0.7	0.1609	ns
Orientation	5.8 ± 0.5	5.9 ± 0.3	0.1609	ns

Means ± SD, *P < 0.05, ****P < 0.0001, paired t test.

The multivariate analysis showed that improvement of the seven subsections at 6 months after operation was included as an independent variable, and improvement of MoCA scores was the dependent variable (Table 5). Our results suggested that all the improvement of Visuospatial/Executive, Naming, Language, and Abstraction had moderate correlation with MoCA improvement. Furthermore, improvement of Visuospatial/Executive, Naming, Language, and Abstraction was associated with MoCA improvement.

**TABLE 5:** Multivariate analysis for improvement of the total MoCA scores and the seven subsection scores.

	Multivariate analysis	
	
	Pearson correlation (r)	*P* value (one-tailed)
Visuospatial/Executive gain	0.4838	0.0039
Naming gain	0.4475	0.0075
Memory and delayed recall gain	0.2052	0.1428
Attention gain	0.2604	0.0862
Language gain	0.4080	0.0140
Abstraction gain	0.5399	0.0013
Orientation gain	0.2376	0.1073

*The improvement of the seven subsections 6 months after operation were included as independent variables, and improvement of MoCA scores was the dependent variable. Subsections gain (Visuospatial/Executive gain, Naming gain, Memory and Delayed recall gain, Attention gain, Language gain, Abstraction gain, Orientation gain): postoperative subsection score – preoperative subsection score. p < 0.025 was considered statistically significant (one tail test).*

According to the results of paired *t*-test, it was found that the subsections of Language and Visuospatial/Executive were associated with the total MoCA improvement.

### The Preoperative and Postoperative Glasgow Benefit Inventory Scores Were Significantly Different

The GBI assessment was performed 6 months after operation. The average benefit at 6 months after operation was found to be +22.8 ± 16, suggesting that the overall quality of life of the patients was significantly improved after operation. The three subscales of GBI were analyzed separately, with general benefit of +21.4 ± 21.1, social benefit of 40.8 ± 28.4, and physical benefit of 10.3 ± 13.7.

### Hearing Improvement Was Correlated With Cognitive Improvement and Improvement of Quality of Life

To explore whether cognitive improvement or improvement of quality of life is correlated with hearing improvement, Pearson’s correlation analysis was performed. As shown in Table 4, a significant correlation between hearing improvement and the increase in MoCA scores was revealed (Table 6). A correlation between hearing improvement and GBI scores was also indicated (Table 6).

**TABLE 6:** Correlation coefficients between hearing improvement and cognitive improvement/quality of life improvement in the present study.

	Correlation coefficient (r)	
	
	MoCA gain	GBI scores
Hearing gain	0.546	0.603
SDSmax gain	0.760	0.840

*Pearson’s correlation analysis. Hearing gain, postoperative ABG-preoperative ABG; SDSmax gain, postoperative SDSmax – preoperative SDSmax; MoCA gain, postoperative total MoCA score-preoperative total MoCA score.*

## Discussion

In the present study, it was found that cognitive functions were improved after tympanoplasty for ARHL and COM. ARHL is a highly prevalent disability in later life, affecting the cognitive capacity of the elderly and even leading to dementia, which has posed a serious threat to quality of life (Griffiths et al., 2020). Furthermore, when ARHL is combined with COM, hearing of the patients will be worse, and it is quite inconvenient to wear hearing aids.

Although tympanoplasty and hearing aids can both improve hearing, they are quite different from each other. Fischer et al. (2011) found that nearly two-thirds of the hearing loss patients did not wear hearing aids because of discomfort, cost, inconvenience, etc (van den Brink et al., 1996; Meyer and Hickson, 2012). Tympanoplasty conducted in the present study not only reconstructed the middle ear to a normal anatomical structure to the maximum extent but also relieved the discomfort, such as otorrhea and tinnitus. Whether hearing aids can positively influence cognitive functions of the elderly with hearing impairment is a mooting question. Mulrow et al. (1990) evaluated the cognitive performance of 188 hearing loss patients who received hearing aids or not. During a 4-month follow-up, it was concluded that cognitive functions were significantly improved in patients receiving hearing aids compared with those assigned to the waiting list (Mulrow et al., 1990). However, in a multicenter double-blind randomized placebo-controlled trial conducted by Nguyen et al. (2017), 51 hearing loss patients with Alzheimer’s disease (AD) were followed up for 6 months to determine the cognitive benefit of hearing aids. The change from baseline of the Alzheimer’s Disease Assessment Scale—Cognitive subscale was assessed, and it was found that cognitive functions were not improved by hearing aids (Nguyen et al., 2017). Although many studies have proven that hearing loss is associated with cognitive decline, it is difficult to obtain a consistent relationship between the use of hearing aids and cognitive improvement in ARHL patients. Nevertheless, the results of our study may explain this paradox.

In the current study, after a 6-month follow-up, it was found that the cognitive functions, especially the language capacity, were significantly improved in the ARHL patients with COM after tympanoplasty. The main difference between hearing aids and surgeries is that hearing improvement is lasting for the patients receiving surgeries, while it is discontinuous for hearing aid users (Fischer et al., 2011). Until now, the underlying mechanisms related to hearing loss and cognitive decline are unclear, and there are various hypotheses (Lin and Albert, 2014; Fulton et al., 2015; Wayne and Johnsrude, 2015; Stahl, 2017), such as cognitive load hypothesis, common cause hypothesis, and cascade hypothesis. Our study supports the cascade hypothesis (Uchida et al., 2019). The cascade hypothesis pointed out that the impoverished auditory signals can cause brain neuropathological alterations (Xie, 2016; Park et al., 2018); thus, constant hearing input is necessary for hearing loss patients to maintain or restore cognitive functions. In our study, by surgeries, the hearing stimulations are lasting for the patients, and thus, the cognition functions are significantly improved. However, the cognitive benefit is uncertain for hearing aid users because of the discontinuous hearing improvement. The same explanation may also apply to cochlear implantation, in which the hearing signals are discontinued while resting or swimming.

Furthermore, we also assessed the quality of life of the patients at 6 months after operation by Glasgow Benefit Inventory, a well-established validated questionnaire used to measure the results of surgical interventions. GBI is widely used to assess the QoL of the patients after ENT surgeries with good reliability. Furthermore, GBI, Chinese version, has also been evaluated by Yang et al. (2020), with good validity and reliability. Previous studies have shown that poor social relationships are a risk factor for incident dementia in later life (Kuiper et al., 2015; Livingston et al., 2017). In our study, patients showed a significantly positive benefit after tympanoplasty, with scores of more than +22.8, especially in social support scores (+40.8 ± 28.4). The results proved that hearing improvement improved social interactions, which may prevent cognitive decline (Hsiao et al., 2018; Qiu and Fratiglioni, 2018).

Therefore, for ARHL patients with COM, active surgical intervention for COM is recommended because hearing improvement may result in cognitive improvement and improvement of quality of life. Because tympanoplasty is performed under general anesthesia, surgical complications seem to be the greatest concern. Vartiainen and Karjalainen (1985) illustrated that no severe complications occurred in the perioperative period of patients aged 60 years. In our study, there were also no severe complications found perioperatively.

Although it was found that tympanoplasty could improve both cognitive functions and quality of life, some limitations must be considered in the present study. First, the sample size in the study was relatively small. However, as there are few similar studies, it adds important knowledge to the current field. Second, the current research focused on the relationship between hearing improvement after middle ear surgeries and cognitive functions/quality of life. The correlation between relief of other discomforts and cognitive functions, such as otorrhea and tinnitus, was not studied. In the future studies, we will conduct COM-specific questionnaires (Wang et al., 2003; Yang et al., 2020) on the patients and further explore the relationships between them.

## Data Availability Statement

The raw data supporting the conclusions of this article will be made available by the authors, without undue reservation.

## Ethics Statement

The studies involving human participants were reviewed and approved by the Ethics Committee of Beijing Tsinghua Changgung Hospital. The patients/participants provided their written informed consent to participate in this study.

## Author Contributions

HY conceptualized and designed the study and approved the final version of the manuscript. JG prepared the draft of the manuscript and made revisions. JC followed up of patients and handled the acquisition of the data. JX and SL performed the analysis and interpretation of the data. All authors contributed to the article and approved the submitted version.

## Conflict of Interest

The authors declare that the research was conducted in the absence of any commercial or financial relationships that could be construed as a potential conflict of interest.

## Publisher’s Note

All claims expressed in this article are solely those of the authors and do not necessarily represent those of their affiliated organizations, or those of the publisher, the editors and the reviewers. Any product that may be evaluated in this article, or claim that may be made by its manufacturer, is not guaranteed or endorsed by the publisher.
